# Chemotherapy-Induced Nausea and Vomiting in Patients With Breast Cancer: Risk Factor and Predictive Model Using Classification and Regression Tree (CART)

**DOI:** 10.7759/cureus.44438

**Published:** 2023-08-31

**Authors:** Bryant Ng, Yufi Kartika Astari, Juan Adrian Wiranata, Benedreky Leo, Susanna H Hutajulu, Mardiah S Hardianti, Kartika W Taroeno-Hariadi, Johan Kurnianda, Ibnu Purwanto

**Affiliations:** 1 Medicine Study Program, Faculty of Medicine, Public Health and Nursing, Universitas Gadjah Mada, Yogyakarta, IDN; 2 Research Scholar, Division of Hematology and Medical Oncology, Department of Internal Medicine, Dr. Sardjito General Hospital, Yogyakarta, IDN; 3 Clinical Epidemiology Program, Master of Clinical Medicine Postgraduate Program, Faculty of Medicine, Public Health and Nursing, Universitas Gadjah Mada, Yogyakarta, IDN; 4 Specialty Program in Internal Medicine, Department of Internal Medicine, Faculty of Medicine, Public Health and Nursing, Universitas Gadjah Mada, Yogyakarta, IDN; 5 Division of Hematology and Medical Oncology, Department of Internal Medicine, Faculty of Medicine, Public Health and Nursing, Universitas Gadjah Mada/Dr. Sardjito General Hospital, Yogyakarta, IDN

**Keywords:** vomiting, nausea, patient reported outcome measures, antineoplastic agents, breast neoplasms

## Abstract

Introduction

Chemotherapy-induced nausea and vomiting (CINV) is a common and debilitating adverse effect of breast cancer chemotherapy. The incidence of CINV in the first cycle of chemotherapy is essential, as it sets the tone for anticipatory CINV and the overall patients' treatment experience. We aimed to investigate the risk factors of first cycle CINV in breast cancer patients and to develop a classification and regression tree (CART) model to predict its occurrence.

Methods

This is a cross-sectional study that nested in a prospective cohort. One hundred and thirty-seven female breast cancer patients receiving highly emetogenic chemotherapy were included. We used the Common Toxicity Criteria for Adverse Events (CTCAE) version 4.0 to assess patient-reported nausea and vomiting in the first chemotherapy cycle. The proportional difference of CINV between sociodemographic and clinicopathologic variables was analyzed using chi-square, and the strength and direction of the relationship with CINV were analyzed using bivariable logistic regression analysis. Multivariable logistic regression and CART analysis included variables with a p-value <0.250.

Results

The incidence of first-cycle CINV was 43.1%. The chi-square test revealed a significant association between insurance status and CINV (p<0.001) and between the stage at diagnosis and CINV (p<0.001). Underweight to normal body mass index (BMI) patients are significantly associated with an increased risk of first-cycle CINV (OR =2.17, 95% CI 1.03-4.56, p =0.041). In hierarchical order, three variables (stage at diagnosis, BMI, and age) were included in the CART model, which significantly influenced the probability of first cycle CINV. With an accuracy of 61.3%, the CART model had a sensitivity of 28.8%, a specificity of 85.9%, a positive predictive value of 60.7%, a negative predictive value of 61.5%, and an area under curve (AUC) of 0.602.

Conclusion

Breast cancer patients with an underweight to normal BMI have a higher risk of developing first-cycle CINV. Our CART model was better at identifying patients who would not develop CINV than those who would. The CART model may provide a simple and effective way to individualize patient care for first-cycle CINV.

## Introduction

Breast cancer is one of the most prevalent and life-threatening malignancies affecting women worldwide. In Indonesia, breast cancer ranks first in incidence, accounts for 30.9% of the total cancer incidence in women and contributed to 11% of cancer-related deaths in 2020 [1]. In Yogyakarta Province, Indonesia, breast cancer cases were dominated by relatively young individuals and those with advanced stages. The local patients had a poor five-year overall survival rate of 51% [2].

Chemotherapy remains a foundation for treating breast cancer but often comes with a range of distressing toxicities, including chemotherapy-induced nausea and vomiting (CINV). Chemotherapy regimens are often categorized based on their emetogenicity, which refers to the drug's potential to induce CINV [3]. CINV is among the most distressing and common non-hematologic toxicities reported by breast cancer patients undergoing chemotherapy [4].

Managing CINV in breast cancer patients is essential due to its significant impact on multiple aspects of a patient's treatment experience. CINV may lead to dehydration, electrolyte imbalances, and malnutrition, potentially resulting in treatment interruptions and dose reductions, compromising the efficacy of chemotherapy given [5]. The intense physical and emotional distress caused by CINV can also severely affect patients' quality of life, with increased anxiety, depression, and diminished social functioning [6], leading to poor treatment adherence [7].

The incidence of CINV in the first cycle of chemotherapy is essential as it sets the tone for anticipatory CINV, which refers to a learned or conditioned response in those who have experienced nausea and vomiting during the previous chemotherapy cycle, resulting in CINV in further cycles. Ensuring the management of CINV during the first cycle of chemotherapy is crucial to stopping anticipatory CINV from occurring, which has also been demonstrated in the previous study [8,9]. The incidence of CINV in the first cycle can also lead to heightened fear and anxiety about experiencing further cycles of CINV, hindering patients' ability to cope effectively with their diagnosis and treatment journey and leading to poor treatment adherence [7,10].

Knowledge of the incidence and severity of CINV is still based on reporting from clinical trials, which may not reflect the reality of CINV incidence and severity in routine clinical practice. Symptoms reported by clinicians in clinical trials often do not report the exact frequency and severity of symptoms subjectively experienced by patients [11]. Recording CINV based on patient-reported outcomes allows patients to precisely describe the symptoms experienced, avoid observer bias, and increase patient-clinician communication, allowing faster and more accurate management [12].

Previous studies have investigated that several factors might affect the incidence of CINV in breast cancer patients undergoing first-cycle chemotherapy, including age, BMI, chemotherapy regimen, serum albumin, Eastern Cooperative Oncology Group (ECOG) Performance Status, number of parity, insurance status, history of motion sickness, and diabetes mellitus [13-17]. Few investigations regarding predictors of CINV in breast cancer patients have been conducted in Indonesia. A case-control study from Indonesia has found that a history of contraception use is associated with late-onset CINV [18]. There is a large gap in the knowledge of predictors of CINV in our local patients with breast cancer, and no study in Indonesia has explored the burden of CINV in the first cycle of chemotherapy. Further, numerous statistical approaches for exploring the predictors of CINV have been used, and regression analysis is one of the most frequently utilized models for its simple and efficient interpretation. Linear regression models assume linear relationships between predictors and outcome variables [19]. However, the occurrence of CINV has a complex and multifactorial underlying mechanism, and the influences of many factors often cause the relationship to be non-linear [8]. The CART methodology has been proposed to overcome the shortcomings of linear regression models and to account for the non-linear relationships between dependent and independent variables.

The CART algorithm generates a decision tree model that is easy to interpret without requiring extensive statistical knowledge. This model provides a visual flow that can be followed and used without a computational interface. Compared to other machine learning models, CART models are more interpretable and readily usable, allowing a more straightforward interpretation of the results [20]. To our knowledge, no study has explored the predictors of first-cycle CINV using CART analysis.

Results derived from this study may lead to recommendations for identifying and managing patients with breast cancer susceptible to first-cycle CINV and increasing the overall treatment experience. Using data from patient-reported outcomes, our study aimed to identify the incidence of CINV in patients receiving highly emetogenic chemotherapy regimens and investigate local sociodemographic and clinical predictors for CINV with the application of CART analysis.

## Materials and methods

This cross-sectional study included patients recruited from a prospective study investigating toxicities in breast cancer chemotherapy and targeting a minimum of 200 patients between 2008 and 2022. Subjects included in this study are histopathologically confirmed female breast cancer patients aged 18 or older who undergo first-line chemotherapy with highly emetogenic regimens in the Hematology and Medical Oncology Division, “Tulip”/Integrated Cancer Clinic, Dr. Sardjito General Hospital, Yogyakarta, Indonesia. The aforementioned prospective study has gained authorization from the joint ethics committee of the Faculty of Medicine, Public Health, and Nursing, Universitas Gadjah Mada/Dr. Sardjito Hospital, Yogyakarta (reference number: KE/FK/0417/EC/2018).

The data for CINV were abstracted from the parent study database. In the main study, all recruited participants were interviewed one and two weeks after the first chemotherapy cycle by a trained research team to gain data on the incidence and severity of chemotherapy toxicities, including CINV. The symptoms recording is based on the translated and validated CTCAE version 4.0 in Bahasa Indonesia, developed by the prospective study research team. Most of the interview sessions were held at the oncology outpatient clinic. However, due to travel restrictions imposed by the COVID-19 pandemic, several subjects' interviews were conducted by phone with the trained research team. CINV was defined as the incidence of nausea concurrent with vomiting at any grade, according to CTCAE version 4.0 [21].

For this study, we included patients who received a highly emetogenic chemotherapy regimen. The emetogenicity of individual chemotherapy agents was classified as low, moderate, and high according to the Multinational Association of Supportive Care in Cancer/European Society for Medical Oncology (MASCC/ESMO) guidelines [22]. The emetogenicity of whole regimens was also classified as low, moderate, and high according to the American Society of Clinical Oncology (ASCO) proposal [23].

Secondary data regarding sociodemographic and clinicopathologic information were also obtained from the prospective study database based on variables known in previous studies associated with CINV. The sociodemographic variables obtained are age, occupation, number of parities, and insurance used for treatment. Age was categorized into ≤60 and >60 years old. The occupation status of the patients was categorized as formal sector workers (those engaged in professional or administrative jobs and often in office settings, including civil servants, lecturers, teachers, and private employees), informal sector workers (those whose work involves manual labor or a skilled trade, including traders, freelancers, farmers, and entrepreneurs), and a group of housewives and retired individuals. Parity was defined as the number of instances in which a woman has given birth to a live neonate at any gestational stage or at 24 weeks or later, irrespective of whether the child was viable or non-viable (such as stillbirths). Parity was then categorized into a group of subjects with less than or equal to two parity and a group of subjects with greater than two parities. Insurance status was categorized into government-incentivized insurance, government non-incentives insurance, and or private insurance. 

The clinicopathologic variables obtained are body mass index (BMI), presenting stage, history of nausea or vomiting one week before chemotherapy, history of diabetes mellitus, and pre-treatment serum albumin level. BMI was categorized into underweight to normal BMI (<23 kg/m2) and an overweight to obese class (BMI ≥23 kg/m2) according to the WHO Asia-Pacific BMI classification. Presenting stage was defined as the subject stage status at diagnosis, recorded according to the seventh edition of the American Joint Committee on Cancer (AJCC) staging system, and divided into stage I to III (non-metastatic) breast cancer and stage IV (metastatic) breast cancer. Information regarding the history of diabetes mellitus and nausea or vomiting before chemotherapy was obtained from the health medical record at the subject's first visit and assessment before commencing chemotherapy. The subject's serum albumin levels were obtained from pre-treatment laboratory examination data, categorized by the median value into ≥4.42 g/dL and <4.42 g/dL.

The patient’s baseline characteristics data were presented as mean and standard deviation (SD) for continuous data and frequency for categorical data. The proportion difference of CINV between sociodemographic and clinicopathologic variables was analyzed using chi-square. A bivariable binary logistic regression test was used to determine the strength and direction of the relationship between sociodemographic and clinicopathologic variables and CINV at the first cycle. Variables with a p-value of <0.250 in the bivariable analyses were further entered into a multivariable binary logistic regression, and a p-value of <0.05 was considered statistically significant.

We used the Classification and Regression Tree (CART) machine learning algorithm from the variables with a p-value <0.250 in the bivariable binary logistic regression analysis to generate an easily interpretable decision tree to predict first-cycle CINV. This algorithm generates nodes containing a group of patients fulfilling the criteria of previous nodes. Each node contains information regarding the estimated probability of the occurrence of CINV, and the proportion of the sample in the node. The CART model was initiated with a single node called the “root node.” This node was recursively divided into subsequent “decision nodes,” with each split maximizing impurity reduction. Impurity, quantified by our model with the Gini index, reflects the extent to which instances of different classes are present in a node. This division continues until the final nodes are generated, termed “leaf nodes”. The stopping criteria in our CART model were the number of observations in a node being less than 20, less than seven observations when generating a leaf node, and a tree depth of 30. The tree was pruned by complexity parameter, which was tuned to maximize accuracy using 10-fold cross-validation, repeated 10 times. A ROC curve analysis was also conducted for the CART model. All statistical analyses were conducted using R statistical software version 4.3.0. The R packages “rpart” [24] and “caret” [25] were used to build the CART model.

## Results

A total of 137 patients with breast cancer who received highly emetogenic regimens were included in the analysis. The mean age of the subjects was 52.1 years. The majority of the subjects have an overweight to obese BMI (59.9%), stage III disease (48.2%), without a history of diabetes mellitus (90.5%), no history of pre-treatment nausea and vomiting (90.5%), have an age less than or equal to 60 years (81.0%), is a housewife or retired (46.0%), informal sector workers (32.8%), has a parity of less than or equal to two (75.2%) and has non-incentivized or private insurance (79.6%). There is a significant association between insurance status and the incidence of first-cycle CINV (p<0.001), with a higher proportion of CINV in the government-incentivized insurance group. A significant association is also present between the stage at diagnosis and the incidence of first-cycle CINV (p<0.001), with a higher proportion of CINV in the non-metastatic group (Table 1).

**Table 1:** Subject’s baseline characteristics and the distribution of CINV in the first cycle of breast cancer chemotherapy (n =137) CINV =chemotherapy-induced nausea and vomiting; SD =Standard Deviation; BMI =Body Mass Index (+) Analyzed using Chi-square; * p <0.05

Variables	Frequency n (%)/mean ± SD			p-value
	All Subjects	No CINV	CINV	
Age (years) (mean ± SD)	52.1 ± 8.8	52.8 +/- 9.8	51.3 ± 7.2	
>60 years	26 (19.0)	18 (23.1)	8 (13.6)	0.160
≤60 years	111 (81.0)	60 (76.9)	51 (86.4)	
Occupation				
Formal sector workers	29 (21.2)	18 (23.1)	11 (18.6)	
Informal sector workers	45 (32.8)	25 (32.1)	20 (33.9)	0.821
Housewife or retired	63 (46.0)	35 (44.9)	28 (47.5)	
Parity				
≤2	103 (75.2)	59 (75.6)	44 (74.6)	0.886
>2	34 (24.8)	19 (24.4)	15 (25.4)	
Insurance status				
Non-incentivized or private	109 (79.6)	63 (80.8)	13 (22.0)	<0.001*
Government-incentivized	28 (20.4)	15 (19.2)	46 (78.0)	
Stage				
IV	27 (19.7)	59 (75.6)	8 (13.6)	<0.001*
I-III	110 (80.3)	19 (24.4)	51 (86.4)	
BMI (WHO Asia-Pacific) (mean ± SD)	24.3 ± 4.7	24.7 +/- 4.6	23.7 ± 4.9	
Overweight to obese (≥23 kg/m^2^)	82 (59.9)	51 (65.4)	31 (52.5)	0.129
Underweight to normal (<23 kg/m^2^)	55 (40.1)	27 (34.6)	28 (47.5)	
Albumin (mean ± SD)	4.3 ± 0.5	4.3 +/- 0.6	4.4 ± 0.5	
<4.42	63 (46.0)	37 (47.4)	26 (44.1)	0.695
≥4.42	74 (54.0)	41 (52.6)	33 (55.9)	
Diabetes mellitus comorbidity				
No	124 (90.5)	69 (88.5)	55 (93.2)	0.518
Yes	13 (9.5)	9 (11.5)	4 (6.8)	
History of nausea or vomiting				
No	124 (90.5)	71 (91.0)	53 (89.8)	0.813
Yes	13 (9.5)	7 (9.0)	6 (10.2)	

In the multivariable binary logistic regression for factors associated with first-cycle CINV, it is shown that patients with underweight to normal BMI are associated with increased risk of CINV in the first cycle (OR =2.17, 95% CI 1.03-4.56, p =0.041) (Table 2).

**Table 2:** Sociodemographic and clinicopathologic factors associated with CINV in the first cycle of breast cancer chemotherapy OR =Odds Ratio; CI =Confidence Interval; Ref =Reference; BMI =Body Mass Index * p < 0.05

Variable	Crude OR (95% CI)	p-value	Adjusted OR (95% CI)	p-value
Age				
>60 years	Ref			
≤60 years	1.91 (0.77-4.76)	0.164	2.32 (0.89-6.05)	0.086
Occupation				
Formal sector workers	Ref		Ref	
Informal sector workers	1.31 (0.50-3.40)	0.580		
Housewife or retired	1.31 (0.53-3.22)	0.557		
Parity				
≤2	Ref		Ref	
>2	1.06 (0.48-2.31)	0.886		
Insurance				
Non-incentivized or private	Ref		Ref	
Government-incentivized	1.19 (0.52-2.73)	0.687		
Stage				
IV	Ref			
I-III	2.05 (0.83-5.09)	0.120	2.53 (0.97–6.58)	0.057
BMI				
Overweight to obese	Ref		Ref	
Underweight to normal	1.71 (0.85-3.41)	0.130	2.17 (1.03-4.56)	0.041*
Albumin (mean ± SD)				
<4.42	Ref		Ref	
≥4.42	0.87 (0.44-1.72)	0.695		
Diabetes mellitus comorbidity				
No	Ref		Ref	
Yes	0.56 (0.16-1.91)	0.352		
History of nausea or vomiting				
No	Ref		Ref	
Yes	1.15 (0.36-3.62)	0.813		

A total of 119 patients (86.9%) developed nausea, 59 patients (43.1%) developed vomiting, and 59 patients (43.1%) developed CINV (both nausea and vomiting) after the first cycle of chemotherapy (Table 3). Further, among 59 patients who developed CINV after the first-cycle of chemotherapy, a total of 31 patients (52.5%) developed CINV in the subsequent cycle, and a total of nine patients (15.3%) developed CINV in more than half of chemotherapy cycles (Table 4).

**Table 3:** Incidence of nausea, vomiting, and CINV throughout the chemotherapy timeline with a highly emetogenic regimen (n =137) CINV =chemotherapy-induced nausea and vomiting

Variables	Timeline of chemotherapy cycles								
	1^st^ cycle n = 137 n (%)	2^nd^ cycle n = 133 n (%)	3^rd^ cycle n = 133 (n (%)	4^th^ cycle n = 130 n (%)	5^th^ cycle n = 117 n (%)	6^th^ cycle n = 117 n (%)	7^th^ cycle n = 88 n (%)	8^th^ cycle n = 85 n (%)	Any cycle n (%)
Nausea	119 (86.9)	106 (79.7)	108 (81.2)	95 (73.1)	48 (41.0)	40 (34.2)	20 (22.7)	24 (28.2)	132 (96.4)
Vomiting	59 (43.1)	46 (34.6)	50 (37.6)	46 (35.4)	25 (21.4)	18 (15.4)	7 (8.0)	5 (5.9)	100 (73.0)
CINV	59 (43.1)	43 (32.3)	49 (36.8)	43 (33.1)	25 (21.4)	15 (12.8)	5 (5.7)	5 (5.9)	100 (73.0)

**Table 4:** Incidence of nausea, vomiting, and CINV throughout chemotherapy with a highly emetogenic regimen in patients who experienced first-cycle CINV (n =59 CINV =chemotherapy-induced nausea and vomiting

Variables	Timeline of chemotherapy cycles							
	2^nd^ cycle n = 56 n (%)	3^rd^ cycle n = 57 n (%)	4^th^ cycle n = 55 n (%)	5^th^ cycle n = 53 n (%)	6^th^ cycle n = 53 n (%)	7^th^ cycle n = 43 n (%)	8^th^ cycle n = 42 n (%)	Any cycle n (%)
Nausea	49 (87.5)	48 (84.2)	45 (81.8)	21 (39.6)	18 (34.0)	8 (18.6)	12 (28.6)	56 (94.9)
Vomiting	33 (58.9)	31 (54.4)	29 (52.7)	11 (20.8)	9 (17.0)	2 (4.7)	4 (9.5)	47 (79.7)
CINV	31 (55.4)	31 (54.4)	27 (49.1)	11 (20.8)	8 (15.1)	1 (2.3)	4 (9.5)	47 (79.7)

The bivariable binary logistic regression analysis shows that the subject's age, BMI, and stage at diagnosis have a p-value of <0.250 and therefore selected as features in the CART algorithm. The CART algorithm included all three predictors in the analysis.

**Figure 1:**
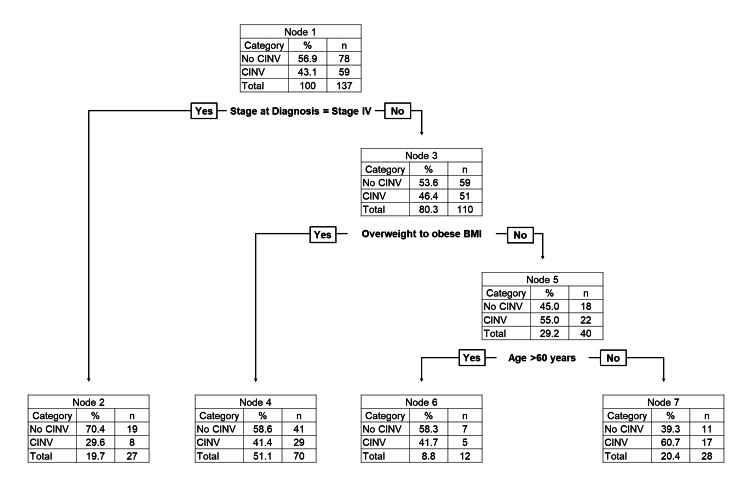
Classification and regression tree (CART) algorithm results of sociodemographic and clinicopathologic factors associated with CINV in the first cycle of breast cancer chemotherapy The CART algorithm generated all three variables included in the analysis and has a depth level of three. The stage at diagnosis in the root node (node 1) was the most decisive variable for discriminating patients with first-cycle CINV. This algorithm further generated two decision nodes (nodes 3 and 5) and four-leaf nodes (nodes 2, 4, 6, and 7), providing first cycle probability based on each division.

The optimal complexity parameter was calculated to be 0.012, and the generated tree has a depth of three. The best discriminator of patients experiencing CINV in the sample was the subject's stage at diagnosis, which was the dividing feature at the root node. At the root node (node 1), there were 137 patients. Most subjects with metastatic disease developed no CINV (node 2; leaf node, 19/27, 70.4%). Subjects with no metastatic disease were directed to node 3. From node 3, 58.6% (41/70) subjects with overweight to obese BMI developed no CINV (node 4; leaf node). On the other hand, patients with underweight to normal BMI were directed to node 5. From node 5, 58.3% (7/12) subjects with age >60 years developed no CINV (node 6; leaf node), whereas 60.7% (17/28) subjects with age ≤60 developed CINV (node 7; leaf node) (Figure 1).

**Figure 2:**
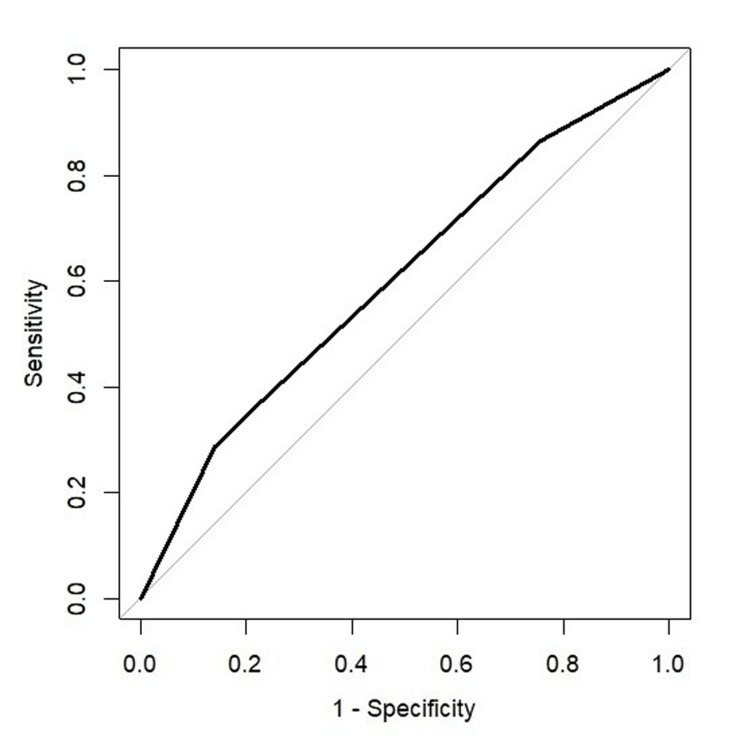
The ROC curve of CART algorithm result ROC curve depicting the performance of the CART algorithm utilizing three variables, yielding an Area Under the Curve (AUC) of 0.602.

A prediction of CINV occurrence was made if the model estimated that the probability of CINV was higher than 50%. Predictions made by this model were compared with the observations. This prediction model has a sensitivity of 28.8%, specificity of 85.9%, positive predictive value of 60.7%, negative predictive value of 61.5%, and accuracy of 61.3%. The ROC curve analysis of the model resulted in an AUC of 0.602 (Figure 2).

## Discussion

As CINV is one of the most occurring non-hematological toxicities in patients with breast cancer receiving chemotherapy [26], the knowledge of predictors of the first-cycle CINV in our local population may improve patient management by identifying susceptible patients and therefore reducing anticipatory CINV and improve overall patient’s treatment experience. To our knowledge, this is the first study in Indonesia which explores risk factors for CINV in the first cycle and applies the CART algorithm, enabling easier interpretation for decision-making. Our findings provide novel insight into the predictors of first-cycle CINV in our local population.

This study utilizes patient-reported outcomes and shows that 43.1% of subjects experience CINV in the first-cycle chemotherapy of highly emetogenic regimens, higher compared to other studies using patient-reported outcomes from the US (25.7%) [13] and Japan (27.3%) [15], China (41.7%) [27], and Malaysia (30.8%) [28]. In our subjects who experienced first-cycle CINV, 52.5% of patients developed CINV in the subsequent cycles, and 15.3% developed CINV in more than half of the chemotherapy cycles. These findings underline the importance of managing CINV in the first cycle in our local population, as also emphasized by previous studies [8,9].

Our analysis found a significantly higher proportion of first-cycle CINV in subjects with government-incentivized insurance status. Previous studies have also found this disparity, indicating that commercial or supplemental insurance patients are more likely to receive guideline-concordant care to assist with their out-of-pocket cost in managing CINV. This disparity highlights the importance of providing optimal care for patients with a lower socioeconomic status that might be burdened by the out-of-pocket cost of managing CINV [29]. However, in our study population, there were no differences between therapeutic regimens based on insurance status. The differences between the proportion of first-cycle CINV based on the insurance status may be influenced by the socioeconomic framework that underlies the insurance status. Further studies are required to investigate this association. We also found that patients with non-metastatic disease were also found to be significantly associated with a higher proportion of first-cycle CINV. Congruent to our finding, a previous study has also found that patients with metastatic disease have a lower risk of developing CINV [27]. Further investigations are warranted to confirm this finding.

Our study found that underweight to normal BMI is associated with first-cycle CINV based on the multivariable logistic regression analysis result. This finding concurs with the previous findings that a lower BMI may be an independent risk factor for CINV [14,16]. However, the mechanisms behind this association between BMI and CINV are unclear. Underlying malnutrition, which may occur more frequently in individuals with a lower BMI, may cause an increased risk of CINV. A study reported that most patients who experienced CINV, which led to decreased dietary consumption, were malnourished [30]. Future investigations to clarify the mechanisms of the link between BMI and CINV may aid in developing strategies to mitigate CINV in individuals with lower BMI.

The CART algorithm generates a decision tree model which is easy to interpret without requiring extensive statistical knowledge. This model provides a visual flow that can be followed and used without a computational interface. Our model had a poor sensitivity (28.8%), good specificity (85.9%), moderate positive predictive value (60.7%) and negative predictive value (61.5%), and an AUC of 0.602. The high specificity means that the model is effective in correctly predicting patients who would not experience CINV, which is beneficial in clinical decision-making. This could allow prioritization of interventions and treatments by not to overprescribe antiemetics in groups with a lower risk of CINV, minimizing cost and allocating resources on patients who are more likely to benefit from preventive measures and treatments. Identifying patients with a lower risk of CINV can also be used to counsel patients and their families about the likelihood of experiencing CINV. Patients with low predicted risk can be reassured about their lower risk, which might alleviate anxiety and improve their overall treatment experience.

In our model, the most decisive predictor of CINV was whether the patient had stage IV cancer. A previous study found that having a clinical stage of stage IV was a significant predictor of CINV in breast cancer patients [27]. The second most decisive factor was whether the patient had an overweight to obese BMI (≥23 kg/m2), which was followed by whether the patient had an age >60 years. Our CART model only has three variables, increasing the ease of use of the model. With good specificity, though with poor sensitivity, our CART model effectively identifies patients at a lower risk of CINV but is not as effective in identifying patients at a higher risk.

The strength of our study includes the prospective collection method for documenting nausea and vomiting. The application of logistic regression analysis and the CART machine learning algorithm for the study endpoint also has provided robust analysis for the study results. However, some limitations also needed to be addressed. First, this study used single-center data, and therefore, interpretation and generalization of the results require careful consideration. Second, the CART model is yet to be prospectively validated in independent samples since we did not divide the data into training and testing datasets. Validation can be done to negate any potential bias in the calculated metrics of the model.

## Conclusions

In the local breast cancer patients receiving first-line chemotherapy, we found that patients with government-incentivized insurance status and a non-metastatic disease have a higher proportion of first-cycle CINV incidence. We also observed that underweight to normal BMI patients have a higher risk of developing first-cycle CINV. From the CART model, we identified that stage at diagnosis, BMI, and age were significant predictors for first-cycle CINV. Our CART model may be an effective and easily interpretable way to categorize patients according to their risk of developing CINV. Though further validation of the model is needed, our CART model allows clinicians to personalize preventive measures, thereby improving patients’ experience during breast cancer chemotherapy.
